# Demographics, Birth Parameters, and Social Determinants of Health Among Opioid-Exposed Mother-Infant Dyads Affected by Neonatal Abstinence Syndrome in Pennsylvania, 2018–2019

**DOI:** 10.1007/s10995-023-03678-5

**Published:** 2023-05-27

**Authors:** Caryn M. Decker, Mohsin Mahar, Callie L. Howells, Zhen-qiang Ma, Carrie Thomas Goetz, Sharon M. Watkins

**Affiliations:** 1 Bureau of Epidemiology, Pennsylvania Department of Health, Harrisburg, PA USA; 2 Office of Drug Surveillance and Misuse Prevention, Pennsylvania Department of Health, Harrisburg, PA USA

**Keywords:** Neonatal abstinence syndrome, Perinatal substance use, Opioids, Social determinants of health

## Abstract

**Objectives:**

To characterize demographics, birth parameters, and social determinants of health among mother-infant dyads affected by neonatal abstinence syndrome (NAS) in Pennsylvania.

**Methods:**

We linked 2018–2019 NAS surveillance data to birth record data using probabilistic methods and then geospatially linked to local social determinants of health data based on residential address. We generated descriptive statistics and used multivariable mixed-effects logistic regression to model the association between maternal characteristics, birth parameters, social determinants of health, and NAS.

**Results:**

In adjusted models maternal age > 24, non-Hispanic white race/ethnicity, low educational attainment, Medicaid as payor at delivery, inadequate or no prenatal care, smoking during pregnancy, and low median household income were associated with NAS. We found no significant associations between NAS and county-level measures of clinician supply, number of substance use treatment facilities, or urban/rural designation.

**Conclusions for Practice:**

This study characterizes mother-infant dyads affected by NAS using linked non-administrative, population data for Pennsylvania. Results demonstrate a social gradient in NAS and inequity in prenatal care receipt among mothers of infants with NAS. Findings may inform implementation of state-based public health interventions.

## Significance

This article is the first to characterize Pennsylvania mother-infant dyads affected by NAS that is not reliant on administrative data. Findings demonstrating a social gradient in NAS and inequity in receipt of care will inform state-based public health practice to better support pregnant people using opioids. Additionally, Pennsylvania was among the first states to initiate NAS surveillance. While data are from 2018 to 2019, they represent the first datasets available, and the methods and limitations described in the article may be instructive to other jurisdictions.

## Introduction

Neonatal abstinence syndrome (NAS) is a constellation of signs and symptoms experienced by newborns exposed to substances such as opioids, benzodiazepines, and barbiturates in utero (CSTE 2019; Finnegan et al., 1975). Over the past two decades, national rates of NAS and maternal opioid use disorder at delivery have been increasing (Haight et al., 2018; Patrick et al., 2012; Winkelman et al., 2018; Hirai et al., 2021). Rates of maternal opioid-related diagnoses per 1,000 delivery hospitalizations increased from 3.5 to 2010 to 8.2 in 2017, and a concomitant increase in rates of NAS birth hospitalization was observed during that period nationally (4.0 in 2010 to 7.3 per 1,000 birth hospitalizations in 2017), and in Pennsylvania (7.5 in 2010 to 14.7 per 1,000 birth hospitalization in 2017) (Hirai et al., 2021). In 2017, the rate of NAS birth hospitalizations in Pennsylvania exceeded the national rate twofold.

As rates of NAS have increased, a growing body of literature has documented the characteristics of affected mother-infant dyads. Studies have found that infants with NAS are more likely to have low birthweight and respiratory complications, contributing to higher rates of NICU admission, prolonged hospital stays, and a higher average cost per stay compared to other birth hospitalizations (Patrick et al., 2012; Winkelman et al., 2018; Hirai et al., 2021). Rates of NAS are also disproportionately higher among low-income mothers and mother-infant dyads covered by Medicaid at delivery (Patrick et al., 2012; Hirai et al., 2021). In recent years, an increasing number of studies have integrated data on local factors such as rural/urban designation, provider availability, and unemployment rates, highlighting the importance of place-based analysis and increased consideration of the influence of social determinants of health (SDOH) on substance use during pregnancy (Villapiano et al., 2017; Faherty et al., 2019; Patrick et al., 2019).

While findings from these existing studies are informative, most studies of NAS have been focused on hospitalization data or other administrative, claims-based databases with only select information available on maternal characteristics and birth parameters. Additional limitations of such data sources include the time lag in the availability of hospitalization data and underestimation of rates of NAS when using administrative datasets (Burns et al., 2007; Jilani et al., 2019). The advent of state-led surveillance of NAS allows for more timely access to epidemiologic data. These data can be linked to other datasets using individual-level identifiers to better characterize state and county-level variation in rates of NAS and inform state-based needs assessment and prevention initiatives (Jilani et al., 2022). Several states have used findings from their surveillance efforts for this purpose (Jilani et al., 2022; Umer et al., 2021).

Accordingly, using surveillance data to characterize the resident population most affected by NAS in Pennsylvania is essential for public health planning and addressing the ongoing impacts of the opioid epidemic. Data from Pennsylvania’s state-led surveillance of opioid-related NAS provides an advantageous alternative to hospitalization data that can provide unique insights into this population. The primary objective of this study was to examine differences in demographics, birth parameters, and SDOH among infants with and without NAS and their mothers to identify modifiable risk factors and inform state-specific public health strategies.

## Methods

We conducted a retrospective, population-based study using two datasets housed by the Pennsylvania Department of Health: 2018–2019 NAS surveillance data and infant birth records. Additional data on zip code and county characteristics were obtained from the Health Resources and Services Administration (HRSA) Area Health Resources Files (AHRF), American Community Survey, and the amfAR Opioid and Health Indicators Database. Pennsylvania NAS surveillance and rapid case reporting began in February 2018 in response to a statewide opioid disaster declaration. The NAS surveillance case definition required reporting of all infants born in-state to Pennsylvania residents between January 10, 2018, and December 31, 2019, who had in-utero exposure to opioids, a NAS diagnosis, and at least one symptom compatible with NAS. Symptoms included in the case definition were body shakes/tremors, seizures/convulsions, overactive reflexes, hypertonia, fussiness, excessive or high-pitched cry, poor feeding/sucking, low weight gain, breathing fast or with difficulty, fever, sweating, blotchy skin, difficulty sleeping or excessive yawning, diarrhea/vomiting, and excessive sneezing.

Cases were reported electronically by Pennsylvania birthing facilities and hospitals within 30 days of diagnosis or discharge and all case reports were assessed by the Department to confirm compliance with surveillance criteria. Information collected on the NAS case report form included maternal and infant names and dates of birth, maternal address, demographics, substance use during the prenatal period, infant laboratory testing for substance exposure, infant symptoms, and infant NAS-related treatment. The Pennsylvania birth record dataset used for linkage included all in-state resident births occurring in calendar years 2018 and 2019. The NAS surveillance dataset and birth records were linked using maternal and infant first, middle, and last names, date of birth, and zip code through the probabilistic linkage software MatchPro. The remaining unlinked resident live births comprised the comparison group. For all plural births, a single record was retained so that each mother was represented only once. The resulting file of cases and controls was linked to publicly available data from HRSA, ACS, and amfAR using maternal zip code or county of residence.

Demographic characteristics, birth parameters, and SDOH were selected for inclusion to analyze both proximal and distal determinants of health and aligned with previously documented associations with NAS (Erwin et al., 2017; Faherty et al., 2019; Lind et al., 2015; Okoroh et al., 2017; Patrick et al., 2019; Villapiano et al., 2017). Infant and maternal characteristics from the birth record included maternal age (≤ 24, 25–29, 30–34, ≥ 35), maternal race and ethnicity (non-Hispanic white, non-Hispanic Black, non-Hispanic Asian or Pacific Islander, Hispanic), maternal educational attainment (high school or below, some college, ≥college graduate), primary payor at delivery (private, public [Medicaid], self-pay), smoking during pregnancy (yes, no), initiation of prenatal care (yes, no), adequacy of prenatal care utilization (inadequate – care started after fourth month of pregnancy and received less than 50% of expected visits, intermediate – care started before fourth month of pregnancy and received 50-79% of expected visits, adequate or adequate plus – care started before fourth month of pregnancy and received ≥ 80% of expected visits), gestational age in weeks (< 37 weeks [pre-term], ≥ 37 weeks [full-term]), birthweight in grams (< 2500 g [low], ≥ 2500 g [normal]), and NICU admission (yes, no). The Adequacy of Prenatal Care Utilization Index was derived from birth record data on timing of prenatal care initiation and number of visits using the established method (Kotelchuck, 1994). Median household income quartile by zip code and county were included from the ACS. The rural/urban designation by county (metropolitan, nonmetropolitan) was included from HRSA’s AHRF and was derived from the 2013 Rural-Urban Continuum classification developed by the United States Department of Agriculture (USDA, 2020) To evaluate the impact of county-level availability of health care providers and substance use treatment facilities, several parameters were used: HRSA’s Mental Health Professional Shortage Area (HPSA) and Primary HPSA designation variables; amFAR’s indicator for number of treatment facilities that provide all three types of medication for opioid use disorder or MOUD (buprenorphine, methadone, naltrexone); and amFAR’s indicator for the number of facilities that provide all three MOUD treatments and accept Medicaid.

Descriptive statistics were used to analyze maternal, infant, and local characteristics on SDOH. To compare characteristics among mother-infant dyads, we used the chi-squared test for categorical variables and the Cochran-Mantel-Haenszel test for ordinal variables. For continuous variables we presented means with standard deviations and assessed differences using the t-test. P < 0.05 was considered statistically significant. For multivariable analysis we generated two multivariable mixed-effects logistic regression models with logistic link to assess associations between predictor variables and NAS as a binary outcome. Both models included random intercepts to control for clustering by location of residence. The first model included county as a random effect and assessed associations between NAS and maternal characteristics, county level measures of median household income by quartiles, healthcare availability, and urban-rural designation. The second model included zip code as a random effect and assessed associations between NAS and maternal characteristics and median household income by quartiles. Measures of association were reported as crude (OR) or adjusted odds ratios (aOR) when controlling for covariates. Analyses were conducted using SAS version 9.4 (SAS Institute). Institutional review board approval was not required as linkage associated with public health surveillance is exempt and subsequent data analysis only required de-identified data.

## Results

Vital records data for 258,182 resident births from 2018 to 2019 were linked to the NAS surveillance dataset. All needed identifiers were available for 3,596 NAS records from 2018 to 2019 and approximately 91% of the NAS case reports (N = 3,271) could be linked to a corresponding record on the birth file. The remaining unlinked resident live births comprised the comparison group (N = 254,911). Table 1 displays demographics, birth parameters, and SDOH among mother-infant dyads by infant NAS status (Table 1). In comparison to non-NAS resident births, a higher percentage of mothers of infants with NAS were non-Hispanic white (87.0% vs. 66.7%, p < 0.001), between the ages of 25 and 29 (35.0% vs. 28.0%, p < 0.001), insured by Medicaid (79.7% vs. 35.0%, p < 0.001), and self-reported smoking during pregnancy (41.2% vs. 9.4%, p < 0.001). A lower percentage of mothers of infants with NAS received any prenatal care (92.4% vs. 98.1%, p < 0.001) or received adequate or adequate plus prenatal care (47.2% vs. 74.1%, p < 0.01) as compared to mothers of infants without NAS. Compared to infants without NAS, infants with NAS were more often preterm (15.9% vs. 8.8%, p < 0.001) or low birthweight (18.6% vs. 7.5%, p < 0.001), and were more likely to be admitted to the NICU (30.6% vs. 8.3%, p < 0.001). Analyses of locality and SDOH revealed that a higher percentage of mothers of infants with NAS resided in nonmetropolitan counties than mothers of infants without NAS (3.5% vs. 2.5%, p < 0.001), in counties designated as mental health HPSAs (19.8% vs. 10.8%, p < 0.001), and in zip codes or counties in the lowest quartile of median household income (36.0% vs. 25.5% for zip code and 29.7% vs. 22.2% for county, p < 0.001). Additionally, mothers of infants with NAS had a lower average number of substance use facilities per county offering all three forms of MOUD (1.9 vs. 2.4, p < 0.001) and a lower number of facilities per county offering all three forms of MOUD that also accepted Medicaid (1.6 vs. 2.0, p < 0.001). Nominal but insignificant differences in county-level primary care HPSA designation were observed between the two populations (p = 0.52).

**Table 1:** Select demographics, birth parameters, and social determinants of health among mother-infant dyads by NAS status of the infant, Pennsylvania 2018–2019

Characteristics	Birth to infant with NAS (N = 3,271)	Other births (N = 254,911)	*P*
	N (%)	N (%)	
**Maternal age**			
≤ 24	393 (12.0)	49,632 (19.5)	< 0.001
25–29	1,145 (35.0)	71,270 (28.0)	
30–34	1,165 (35.6)	81,972 (32.2)	
≥ 35	568 (17.4)	51,763 (20.3)	
Missing	0	274	
**Maternal race/ethnicity**			
Non-Hispanic Asian or Pacific Islander	46 (1.4)	15,485 (6.1)	< 0.001
Non-Hispanic Black	244 (7.6)	36,835 (14.6)	
Hispanic	127 (4.0)	31,510 (12.5)	
Non-Hispanic White	2,791 (87.0)	168,106 (66.7)	
Missing	63	2,975	
**Maternal education**			< 0.001
High school or below	2,112 (66.1)	94,227 (37.4)	
Some college	976 (30.5)	64,349 (25.5)	
Bachelors or above	108 (3.4)	93,564 (37.1)	
Missing	75	2,771	
**Primary payor at delivery**			< 0.001
Private Insurance	493 (15.8)	142,740 (58.0)	
Medicaid	2,481 (79.7)	86,177 (35.0)	
Self-pay	19 (0.6)	11,648 (4.7)	
Other	121 (3.9)	5,529 (2.2)	
Missing	157	8817	
**Initiated prenatal care**			< 0.001
Yes	2,846 (92.4)	241,526 (98.1)	
No	234 (7.6)	4,566 (1.9)	
Missing	191	8,819	
**Adequacy of Prenatal Care Utilization Index**			< 0.01
Adequate or Adequate Plus	1,259 (47.2)	167,424 (74.1)	
Intermediate	362 (13.5)	22,696 (10.0)	
Inadequate	1,049 (39.3)	35,856 (15.9)	
Missing	601	28,935	
**Smoking during pregnancy**			< 0.001
Yes	1,348 (41.2)	24,055 (9.4)	
No	1,922 (58.8)	230,800 (90.6)	
Missing	1	56	
**Infant gestational age**			
Preterm (< 37 weeks)	514 (15.9)	22,290 (8.8)	< 0.001
Term (≥ 37 weeks)	2,709 (84.1)	231,780 (91.2)	
Missing	48	841	
**Infant birthweight**			< 0.001
Low (< 2500 g)	605 (18.6)	18,937 (7.5)	
Normal (≥ 2500 g)	2,649 (81.4)	235,037 (92.5)	
Missing	17	937	
**Infant NICU admission**			< 0.001
Yes	1,002 (30.6)	21,156 (8.3)	
No	2,269 (69.4)	233,755 (91.7)	
**Zip code median household income quartile** ^**a**^			< 0.001
1	1,163 (36.0)	64,911 (25.5)	
2	809 (25.0)	48,874 (19.2)	
3	684 (21.2)	57,870 (22.8)	
4	577 (17.8)	82,433 (32.4)	
Missing	38	823	
**County median household income quartile** ^**b**^			< 0.001
1	970 (29.7)	56,644 (22.2)	
2	557 (17.0)	30,858 (12.1)	
3	815 (24.9)	56,344 (22.1)	
4	926 (28.3)	111,060 (43.6)	
Missing	3	5	
**Urban/rural county of residence**			< 0.001
Metropolitan	3,136 (96.5)	248,254 (97.5)	
Non-metropolitan	114 (3.5)	6,330 (2.5)	
Missing	21	327	
**Mental health HPSA designation**			< 0.001
The whole county designated	646 (19.8)	27,471 (10.8)	
One or more parts of the county designated	2,203 (67.4)	194,499 (76.3)	
None of the county designated	419 (12.8)	32,936 (12.9)	
Missing	3	5	
**Primary care HPSA designation**			0.52
The whole county designated	11 (0.3)	539 (0.2)	
One or more parts of the county designated	2,941 (90.0)	228,385 (89.6)	
None of the county designated	316 (9.7)	25,982 (10.2)	
Missing	3	5	
	Mean (SD)	Mean (SD)	
**Number of substance treatment facilities with all three forms of MOUD in county of residence** ^**c**^	1.9 (3.5)	2.4 (3.7)	< 0.001
**Number of substance treatment facilities with all three forms of MOUD that accept Medicaid in county of residence** ^**c**^	1.6 (2.9)	2.0 (3.1)	< 0.001

Note. NAS = Neonatal Abstinence Syndrome; NICU = Neonatal Intensive Care Unit; HPSA = Health Professional Shortage Area; MOUD = Medication for opioid use disorder

^a^ Median household income by zip code in quartile 1 is ≤$46,250; quartile 2, $46,251-$54,872; quartile 3, $54,873-$66,250; and quartile 4 ≥$66,251

^b^ Median household income by county in quartile 1 is ≤$48,224; quartile 2, $48,225-$51,646; quartile 3, $51,647-$59,713; and quartile 4 ≥$59,714

^c^ Three forms of MOUD provided are buprenorphine, methadone, and naltrexone

Tables 2 and 3 show results from the unadjusted and adjusted multivariable mixed-effects logistic regression models (Tables 2 and 3). After controlling for county and fixed effects, maternal age (aOR = 3.0; 95% CI = 2.7, 3.4 for 25–29; aOR = 4.4; 95% CI = 3.9, 5.0 for 30–34, aOR = 3.7; 95% CI = 3.3, 4.3 for ≥ 35, as compared to ≤ 24), maternal race/ethnicity (non-Hispanic white vs. non-Hispanic Black aOR = 6.0; 95% CI = 5.2, 7.0), primary payor at delivery (aOR = 4.9, 95% CI = 4.4, 5.5 for Medicaid and aOR = 0.1, 95% CI = 0.1, 0.2 for self-pay, as compared to private insurance), and smoking during pregnancy (aOR = 3.3; 95% CI = 3.1, 3.6) were significant predictors of NAS as an outcome. Educational attainment (aOR = 0.7; 95% CI = 0.7, 0.8 for some college and aOR = 0.1; 95% CI = 0.1, 0.1 for ≥ BS, respectively, as compared to ≤ HS) and receipt of prenatal care (aOR = 1.8; 95% CI = 1.6, 2.2 for no prenatal care vs. any prenatal care; aOR = 2.9; 95% CI = 2.6, 3.2 for inadequate utilization of prenatal care, aOR = 2.2; 95% CI = 2.0, 2.5 for intermediate prenatal care utilization; as compared to adequate or adequate plus prenatal care utilization) appeared to be protective. These associations were similar in direction and magnitude in the model that adjusted for zip code of residence (Table 3). Additionally, residents in zip codes with a household median income in the lowest quartile had 40% higher odds of having an infant with NAS as compared to those residing in a zip code in the highest quartile (aOR = 1.4; 95% CI = 1.2,1.6), although the same association was not observed in the first model that assessed this by county. No significant association was observed between NAS and residence in a metropolitan vs. non-metropolitan county, residence in a county with a mental health or primary health HPSA designation, the number of treatment facilities, or the number of treatment facilities accepting Medicaid.

**Table 2:** Maternal demographics, birth parameters, and county social determinants of health in association with NAS in Pennsylvania, 2018–2019

	NAS	
**Characteristic**	**OR (95% CI)**	**AOR**^**a**^ **(95% CI)**
**Maternal age**		
≤ 24	REF	REF
25–29	2.2 (1.9, 2.4)	3.0 (2.7, 3.4)
30–34	2.1 (1.9, 2.3)	4.4 (3.9, 5.0)
≥ 35	1.7 (1.5, 1.9)	3.7 (3.3, 4.3)
**Maternal race/ethnicity**		
Non-Hispanic Asian or Pacific Islander	0.5 (0.4, 0.7)	1.0 (0.7, 1.2)
Non-Hispanic Black	REF	REF
Hispanic	0.7 (0.6, 0.9)	0.9 (0.7, 1.1)
Non-Hispanic White	2.5 (2.2, 2.9)	6.0 (5.2, 7.0)
**Maternal education**		
High school or below	REF	REF
Some college	0.7 (0.6, 0.7)	0.7 (0.7, 0.8)
Bachelors or above	0.1 (0.1, 0.1)	0.1 (0.1, 0.1)
**Primary payor at delivery**		
Private insurance	REF	REF
Medicaid	8.5 (7.7, 9.4)	4.9 (4.4, 5.5)
Self-pay	0.4 (0.3, 0.7)	0.1 (0.1, 0.2)
**Initiated prenatal care**		
No	5.1 (4.4, 5.9)	1.8 (1.6, 2.2)
Yes	REF	REF
**Adequacy of Prenatal Care Utilization Index**		
Inadequate	4.1 (3.7, 4.4)	2.9 (2.6, 3.2)
Intermediate	2.3 (2.1, 2.6)	2.2 (2.0, 2.5)
Adequate or Adequate Plus	REF	REF
**Any smoking during pregnancy**		
No	REF	REF
Yes	6.0 (5.6, 6.5)	3.3 (3.1, 3.6)
**County median household income quartile** ^**b**^		
1	2.8 (1.7, 4.5)	1.8 (1.0, 3.0)
2	2.0 (1.3, 3.3)	1.2 (0.7, 1.9)
3	1.9 (1.2, 3.1)	1.3 (0.9, 2.1)
4	REF	REF
**Urban/rural designation for county of residence**		
Metropolitan	1.1 (0.9, 1.4)	1.2 (1.0, 1.5)
Non-metropolitan	REF	REF
**Mental health HPSA designation in county of residence**		
None of the county designated	REF	REF
One or more parts of the county designated	1.1 (0.6, 1.8)	0.9 (0.5, 1.5)
The whole county designated	1.7 (1.0, 2.9)	0.8 (0.5, 1.5)
**Primary care provider HPSA designation in county of residence**		
None of the county designated	REF	REF
One or more parts of the county designated	1.3 (0.8, 2.3)	1.2 (0.7, 2.2)
The whole county designated	1.7 (0.3, 9.3)	1.9 (0.5, 7.5)
**Number of substance treatment facilities providing all three forms of MOUD** ^**c**^	0.9 (0.8, 1.0)	0.9 (0.4, 2.1)
**Number of substance treatment facilities providing all three forms of MOUD that accept Medicaid** ^**c**^	0.9 (0.8, 1.0)	1.1 (0.4, 2.7)

Note. NAS = Neonatal Abstinence Syndrome; AOR = adjusted odds ratio; CI = confidence interval; REF = referent group; HPSA = Health Professional Shortage Area, MOUD = Medication for opioid use disorder

^**a**^ Adjusted for all covariates listed and with random intercept included to control for clustering by maternal county of residence

^b^ Median household income by county in quartile 1 is ≤$48,224; quartile 2, $48,225-$51,646; quartile 3, $51,647-$59,713; and quartile 4 ≥$59,714

^c^ Three forms of MOUD provided are buprenorphine, methadone, and naltrexone

**Table 3:** Maternal demographics, birth parameters, and zip code social determinants of health in association with NAS in Pennsylvania, 2018–2019

	NAS	
Characteristic	OR (95% CI)	AOR^a^ (95% CI)
**Maternal Age**		
≤ 24	REF	REF
25–29	2.2 (2.0, 2.5)	3.0 (2.7, 3.4)
30–34	2.2 (2.0, 2.5)	4.4 (3.9, 5.0)
≥ 35	1.8 (1.6, 2.1)	3.7 (3.2, 4.3)
**Maternal race/ethnicity**		
Non-Hispanic Asian or Pacific Islander	0.7 (0.5, 0.9)	1.0 (0.7, 1.4)
Non-Hispanic Black	REF	REF
Hispanic	0.7 (0.6, 0.9)	0.9 (0.7, 1.1)
Non-Hispanic White	3.6 (3.1, 4.2)	6.7 (5.8, 7.8)
**Maternal education**		
High school or below	REF	REF
Some college	0.7 (0.6, 0.7)	0.7 (0.7, 0.8)
Bachelors or above	0.1 (0.1, 0.1)	0.1 (0.1, 0.1)
**Primary payor at delivery**		
Private insurance	REF	REF
Medicaid	8.9 (8.0, 9.8)	4.8 (4.3, 5.4)
Self-pay	0.4 (0.3, 0.7)	0.1 (0.1, 0.2)
**Initiated prenatal care**		
No	4.8 (4.1, 5.6)	1.8 (1.6, 2.2)
Yes	REF	REF
**Adequacy of Prenatal Care Utilization Index**		
Inadequate	4.1 (3.8, 4.5)	2.9 (2.6, 3.2)
Intermediate	2.3 (2.1, 2.6)	2.2 (1.9, 2.5)
Adequate or Adequate Plus	REF	REF
**Any Smoking During Pregnancy**		
No		REF
Yes	5.9 (5.5, 6.4)	3.3 (3.0, 3.5)
**Zip code median household income quartile** ^**b**^		
1	3.1 (2.6, 3.8)	1.4 (1.2, 1.6)
2	2.6 (2.1, 3.1)	1.2 (1.0, 1.4)
3	1.8 (1.5, 2.2)	1.0 (0.8, 1.2)
4	REF	REF

Note. NAS = Neonatal Abstinence Syndrome; AOR = adjusted odds ratio; CI = confidence interval; REF = referent group

^**a**^ Adjusted for all covariates listed and with random intercept included to control for clustering by maternal zip code of residence

^b^ Median household income by zip code in quartile 1 is ≤$46,250; quartile 2, $46,251-$54,872; quartile 3, $54,873-$66,250; and quartile 4 ≥$66,251

## Discussion

This study provides comparative insight on SDOH and maternal and infant characteristics among mother-infant dyads affected by NAS in 2018-2019 using population-level surveillance data. Overall, mothers of opioid-exposed infants with NAS were more often between the ages of 25 and 34 and identified as non-Hispanic white than mothers of infants without NAS, which is consistent with prevailing literature (Okoroh et al., 2017; Lind et al., 2015; Erwin et al., 2017). Observed differences in opioid use disorder by race and ethnicity have been attributed to differential prescribing (Peeler et al., 2020). Historically, people identifying as non-Hispanic white were more likely to receive an opioid prescription to treat pain than people identifying as Black or other races in the United States due to racial bias (Harrison et al., 2018; Hoffman et al., 2016; Santoro et al. 2018). Differential prescribing has contributed to higher rates of opioid use disorder among white women and may also contribute to racial/ethnic differences in NAS diagnoses (Peeler et al., 2020; Harrison et al., 2018). While the Black-white gap in opioid prescribing for pain and prescription misuse appears to have narrowed nationally in recent years, differential access to MOUD persists (Harrison et al., 2018; Schuler et al., 2021). Recent studies of Medicaid-enrolled pregnant people in Pennsylvania have demonstrated that MOUD was more likely to be received by non-Hispanic white pregnant women than non-Hispanic Black or Hispanic women (Krans et al., 2019; Gao et al., 2022).

Findings in the present study also demonstrate that mothers of infants with NAS were less likely to have initiated prenatal care or received adequate prenatal care than mothers of infants without NAS. This is notable because nearly 80% of women who gave birth to an infant with NAS had Medicaid at delivery and prenatal care is a covered benefit. Screening for substance use and co-morbidities should occur universally upon initiation of prenatal care, making it a key opportunity for birthing people to connect to the healthcare system and receive both education and referrals to treatment (ACOG, 2017). Prior research suggests that barriers to entry into prenatal care among women using substances may include lack of transportation, mistrust of the medical system, and fear of stigma or legal implications (Frazer et al., 2019). These barriers highlight the importance of trauma-informed, supportive care from unbiased providers throughout the perinatal period (Renbarger et al., 2022). Strategies that promote comprehensive prenatal care, address barriers and concerns, and allow for honest communication about substance use with a trusted provider should be prioritized.

The results from the present study also reinforce prior studies at the national level and in other jurisdictions which suggest a social gradient in NAS as mothers of infants with NAS were more likely to reside in a zip code with a low household annual income, be Medicaid enrolled, or have lower educational attainment than their counterparts in Pennsylvania who gave birth to an infant without NAS (Hirai et al., 2021; Umer et al., 2021; Lake et al., 2021). Economic instability and the “cumulative disadvantage” of poverty and lack of opportunity have been posited as risk factors for substance use and misuse (Patrick et al., 2019; Zoorob et al., 2017). While the associations between economic insecurity, substance use, and adverse opioid-related outcomes are multifactorial, social and structural inequities are important root causes of the opioid epidemic and related health outcomes (Patrick et al., 2019; Zoorob et al., 2017; Dasgupta et al., 2018). Findings from this study suggest continued focus on strategies that advance health equity and reduce income and opportunity inequalities is merited. Smoking during pregnancy was also more common among mothers of infants with NAS than mothers who gave birth to an infant without NAS, which has also been observed elsewhere (Erwin et al., 2017; Metz et al., 2018).

Notably, associations between NAS and HPSA designations for mental or primary health care at the county-level and NAS were not significant in multivariable analysis despite prior findings suggesting an association between mental HPSA designation and rates of NAS in select counties at the national level (Patrick et al., 2019). Patrick et al. (2019) theorized that their observed association may have been due to increased risk of opioid use among those with untreated mental health issues. However, the Patrick et al. (2019) study was also ecological and did not include or adjust for patient-level characteristics, which may explain the differing results. Given that multiple factors influence whether a person seeks or receives adequate preventive, mental, or preconception care, county-level HPSA designations may be too rudimentary to adequately measure healthcare access. Finally, despite literature suggesting that national rates of opioid-related deliveries and NAS have been increasing disproportionately in rural counties and areas, the present study did not find a significant association between NAS and residence in a rural or urban county in Pennsylvania in multivariable analysis (Villapiano et al., 2017). However, incidence rates of NAS in the state remain highest in less populous regions of the state, suggesting that the differential impact of opioids and NAS on communities across Pennsylvania may not have been not captured using the county-level variable (Pennsylvania Department of Health 2021).

There are several important limitations of this study. First, an assessment of Pennsylvania’s NAS surveillance during 2018 and 2019 suggests that not all cases of NAS were reported to the Department (Krause et al., 2021). The inclusion of “NAS diagnosis” in the case definition may have contributed to underreporting as diagnosis is subjective based on facility criteria. While incorporating clinician diagnosis of NAS into the NAS case definition is common among other states with active surveillance (Jilani et al., 2022), it is possible that this criterion may have resulted in misclassification in the present study. Further, during 2018–2019 the Department only collected data on cases of NAS resulting from opioid exposure. Accordingly, this study did not consider infants who experienced withdrawal due to other substances, such as benzodiazepines or barbiturates. We also relied upon facilities to report accurate and timely case information. Data quality issues, including duplicate case reports and missing data on identifiers needed for linkage, required us to exclude nearly 4% of reported NAS cases during 2018-2019 from the analysis. Another limitation is that during the nascent stage of surveillance, data collection was limited to the most important parameters to facilitate participation in the new, rapid reporting process. We therefore did not collect information on whether mothers were using illicit or prescribed opioids or receiving MOUD during pregnancy. To address this limitation, future studies by this team will incorporate Prescription Drug Monitoring Program (PDMP) data to further characterize prescribing patterns before, during, and after pregnancy. Incorporation of data from the PDMP will also make it possible to identify and assess mother-infant dyads exposed to opioids during pregnancy that did not meet the NAS surveillance case definition. Finally, throughout this paper we use the words mother and maternal when describing birthing people. We acknowledge the limitations of this approach as not all pregnant or birthing people identify as women.

Despite these limitations, the study had several strengths including a large sample size of over 3,000 NAS births, linkage to the birth record for comparison, and availability of individual-level maternal and infant characteristics. The results from this study demonstrate that characteristics of the mother-infant dyads affected by NAS during 2018-2019 in Pennsylvania are similar to those observed elsewhere. System-level and social determinants of health, including access to prenatal care and economic stability, are important predictors of opioid use during pregnancy and are associated with NAS as an outcome in Pennsylvania. These findings suggest that multi-level strategies which address both economic and social policies that perpetuate bias and stigma and individual-level factors, such as smoking and entry into prenatal care, are warranted in Pennsylvania.

## Conclusions for Practice

The use of linked, population-level, non-administrative NAS data with birth data provided an opportunity to further describe the demographics, birth parameters, and SDOH of mother-infant dyads affected by NAS in Pennsylvania. Findings from this paper highlight the important role of social factors in determining opioid-related health outcomes, including NAS, and provide a basis for future analysis incorporating Prescription Drug Monitoring Program data on prescribing patterns for people of child-bearing age. Given the lack of a standardized national surveillance system for NAS, results of state-led surveillance fill a critical data gap. Pennsylvania will continue to leverage surveillance data to characterize the incidence of NAS, understand trends in substance use across the state, and inform public health programming for birthing and pregnant people using substances and their infants. Accordingly, results from this paper may inform state-based needs assessments and implementation of prevention efforts and programming that further center health equity and address the social determinants of health.

## Data Availability

The datasets are not publicly available.

## References

[CR2] Burns L, Mattick RP (2007). Using population data to examine the prevalence and correlates of neonatal abstinence syndrome. Drug and Alcohol Review.

[CR3] Committee opinion no (2017).

[CR4] Council for State and Territorial Epidemiologists (CSTE). (2019). *Neonatal abstinence syndrome standardized case definition*. Council for State and Territorial Epidemiologists. https://cdn.ymaws.com/www.cste.org/resource/resmgr/ps/2019ps/19-MCH-01_NAS_updated_5.7.19.pdf

[CR5] Dasgupta N, Beletsky L, Ciccarone D (2018). Opioid crisis: No easy fix to its social and economic determinants. American Journal of Public Health.

[CR6] Erwin PC, Meschke LL, Ehrlich SF, Lindley LC (2017). Neonatal abstinence syndrome in east Tennessee: Characteristics and risk factors among mothers and infants in one area of Appalachia. Journal of Health Care for the Poor and Underserved.

[CR7] Faherty LJ, Kranz AM, Russell-Fritch J, Patrick SW, Cantor J, Stein BD (2019). Association of punitive and reporting state policies related to substance use in pregnancy with rates of neonatal abstinence syndrome. JAMA Network Open.

[CR8] Finnegan LP, Kron RE, Connaughton JF, Emich JP (1975). Assessment and treatment of abstinence in the infant of the drug-dependent mother. International Journal of Clinical Pharmacology and Biopharmacy.

[CR9] Frazer Z, McConnell K, Jansson LM (2019). Treatment for substance use disorders in pregnant women: Motivators and barriers. Drug and Alcohol Dependence.

[CR10] Gao YA, Drake C, Krans EE, Chen Q, Jarlenski MP (2022). Explaining racial-ethnic disparities in the receipt of medication for opioid use disorder during pregnancy. Journal of Addiction Medicine Publish Ahead of Print.

[CR11] Haight SC, Ko JY, Tong VT, Bohm MK, Callaghan WM (2018). Opioid use disorder documented at delivery hospitalization—United states, 1999–2014. MMWR Morbidity and Mortality Weekly Report.

[CR12] Harrison JM, Lagisetty P, Sites BD, Guo C, Davis MA (2018). Trends in prescription pain medication use by race/ethnicity among US adults with noncancer pain, 2000–2015. American Journal of Public Health.

[CR13] Hirai AH, Ko JY, Owens PL, Stocks C, Patrick SW (2021). Neonatal abstinence syndrome and maternal opioid-related diagnoses in the US, 2010–2017. Obstetrical & Gynecological Survey.

[CR14] Hoffman, K. M., Trawalter, S., Axt, J. R., & Oliver, M. N. (2016). Racial bias in pain assessment and treatment recommendations, and false beliefs about biological differences between Blacks and whites. *Proceedings of the National Academy of Sciences*, *113*(16), 4296–4301. 10.1073/pnas.1516047113 10.1073/pnas.1516047113 PMC4843483 27044069

[CR15] Jilani SM, Frey MT, Pepin D, Jewell T, Jordan M, Miller AM, Robinson M, Mars S, Bryan T, Ko M, Ailes JY, McCord EC, Gilchrist RF, Foster J, Lind S, Culp JN, Penn L, Reefhuis J (2019). Evaluation of state-mandated reporting of neonatal abstinence syndrome—six states, 2013–2017. MMWR Morbidity and Mortality Weekly Report.

[CR16] Jilani SM, West K, Jacobus-Kantor L, Ali MM, Nyakeriga A, Lake-Burger H, Robinson M, Barnes AE, Jewell T, Gallaway S (2022). Evaluation of state-led surveillance of neonatal abstinence syndrome—six U.S. States, 2018–2021. MMWR Morbidity and Mortality Weekly Report.

[CR17] Kotelchuck M (1994). An evaluation of the Kessner adequacy of prenatal care index and a proposed adequacy of prenatal care utilization index. American Journal of Public Health.

[CR18] Krans EE, Kim JY, James AE, Kelley D, Jarlenski MP (2019). Medication-assisted treatment utilization among pregnant women with opioid use disorder. Obstetrics and Gynecology.

[CR19] Krause KH, Gruber JF, Ailes EC, Anderson KN, Fields VL, Hauser K, Howells CL, Longenberger A, McClung N, Oakley LP, Reefhuis J, Honein MA, Watkins SM (2021). Assessment of neonatal abstinence syndrome surveillance—Pennsylvania, 2019. MMWR Morbidity and Mortality Weekly Report.

[CR20] Lake ET, French R, Clark RRS, O’Rourke K, Lorch S (2021). Newborns with neonatal abstinence syndrome are concentrated in poorer-quality hospitals. Hospital Pediatrics.

[CR21] Lind JN, Petersen EE, Lederer PA, Phillips-Bell GS, Perrine CG, Li R, Hudak M, Correia JA, Creanga AA, Sappenfield WM, Curran J, Blackmore C, Watkins SM, Anjohrin S (2015). Infant and maternal characteristics in neonatal abstinence syndrome—selected hospitals in Florida, 2010–2011. Morbidity and Mortality Weekly Report.

[CR22] Metz VE, Brown QL, Martins SS, Palamar JJ (2018). Characteristics of drug use among pregnant women in the United States: Opioid and non-opioid illegal drug use. Drug and Alcohol Dependence.

[CR23] Okoroh EM, Gee RE, Jiang B, McNeil MB, Hardy-Decuir BA, Zapata AL (2017). Neonatal abstinence syndrome: Trend and expenditure in Louisiana Medicaid, 2003–2013. Maternal and Child Health Journal.

[CR25] Patrick, S. W., Schumacher, R. E., Benneyworth, B. D., Krans, E. E., McAllister, J. M., & Davis, M. M. (2012). Neonatal abstinence syndrome and associated health care expenditures: United States, 2000–2009. *Journal Of The American Medical Association*, *307*(18), 10.1001/jama.2012.3951 10.1001/jama.2012.3951 22546608

[CR24] Patrick SW, Faherty LJ, Dick AW, Scott TA, Dudley J, Stein BD (2019). Association among county-level economic factors, clinician supply, metropolitan or rural location, and neonatal abstinence syndrome. Journal Of The American Medical Association.

[CR26] Peeler M, Gupta M, Melvin P, Bryant AS, Diop H, Iverson R, Callaghan K, Wachman EM, Singh R, Houghton M, Greenfield SF, Schiff DM (2020). Racial and ethnic disparities in maternal and infant outcomes among opioid-exposed mother–infant dyads in Massachusetts (2017–2019). American Journal of Public Health.

[CR27] Pennsylvania Department of Health (2021). *Neonatal Abstinence Syndrome: 2019 Report*. https://www.health.pa.gov/topics/Documents/Opioids/2019%20NAS%20REPORT.pdf

[CR28] Renbarger KM, Trainor KE, Place JM, Broadstreet A (2022). Provider characteristics associated with trust when caring for women experiencing substance use disorders in the perinatal period. Journal of Midwifery & Women’s Health.

[CR29] Santoro TN, Santoro JD (2018). Racial bias in the US opioid epidemic: A review of the history of systemic bias and implications for care. Cureus.

[CR30] Schuler MS, Schell TL, Wong EC (2021). Racial/ethnic differences in prescription opioid misuse and heroin use among a national sample, 1999–2018. Drug and Alcohol Dependence.

[CR31] Umer A, Lilly C, Hamilton C, Breyel J, Allen L, Rompala A, Moore C, O’Dierno P, John C (2021). Disparities in neonatal abstinence syndrome and health insurance status: A statewide study using non–claims real-time surveillance data. Paediatric and Perinatal Epidemiology.

[CR32] United States Department of Agriculture. Economic Research Service. Rural-urban continuum codes (2020). Retrieved January 23, 2023, from https://www.ers.usda.gov/data-products/rural-urban-continuum-codes/

[CR33] Villapiano NLG, Winkelman TNA, Kozhimannil KB, Davis MM, Patrick SW (2017). Rural and urban differences in neonatal abstinence syndrome and maternal opioid use, 2004 to 2013. JAMA Pediatrics.

[CR34] Winkelman TNA, Villapiano N, Kozhimannil KB, Davis MM, Patrick SW (2018). Incidence and costs of neonatal abstinence syndrome among infants with Medicaid: 2004–2014. Pediatrics.

[CR35] Zoorob MJ, Salemi JL (2017). Bowling alone, dying together: The role of social capital in mitigating the drug overdose epidemic in the United States. Drug and Alcohol Dependence.

